# Hepaticocystic duct and a rare extra-hepatic "cruciate" arterial anastomosis: a case report

**DOI:** 10.1186/1752-1947-2-37

**Published:** 2008-02-06

**Authors:** Vasitha Abeysuriya, Sujatha Salgado, Kemal I Deen, Sumudu K Kumarage

**Affiliations:** 1 Department of Clinical Anatomy, Faculty of Medicine, University of Kelaniya, Ragama, Sri Lanka; 2 Department of Surgery, Faculty of Medicine, University of Kelaniya, Ragama, Sri Lanka

## Abstract

**Introduction:**

The variations in the morphological characteristics of the extra-hepatic biliary system are interesting.

**Case presentation:**

During the dissection of cadavers to study the morphological characteristics of the extra-hepatic biliary system, a 46-year-old male cadaver was found to have drainage of the common hepatic duct drains directly into the gall bladder neck. The right and left hepatic ducts were not seen extra-hepatically. Further drainage of the bile away from the gallbladder and into the duodenum was provided by the cystic duct. Formation of the common bile duct by the union of the common hepatic duct and cystic duct was absent. Further more the right hepatic artery was found to be communicating with the left hepatic artery by a "bridging artery" after giving rise to the cystic artery. An accessory hepatic artery originated from the "bridging artery" forming a "cruciate" hepatic arterial anastomosis.

**Conclusion:**

Combination of a Hepaticocystic duct and an aberrant variation in the extra-hepatic arterial system is extremely rare.

## Introduction

The variations in the morphological characteristics of the extra-hepatic biliary system are numerous. It has been stated that the extra-hepatic biliary system has more anomalies in one cubic centimeter of the space around the region of the cystic duct than any other part of the body [1,2]. These anomalies add to operative difficulties during cholecystectomy.

The incidence of congenital anomalies of the extra-hepatic biliary system varies between 0.58% and 47.2% [3]. Due to the scarcity of studies of this regional anatomy, the exact incidence of all the anomalies of the biliary system is not known but as it has been observed that, vascular anomalies are more frequent than those of the ductal system [2,4]. Anomalies of the extra-hepatic biliary system can arise from the gallbladder, cystic duct, hepatic ducts or the common bile duct as a result of aberrations of normal embryological development. Therefore, it is essential to appreciate the extent of anomalies of the extra-hepatic biliary system.

One such rare anomaly is where the right and left hepatic ducts are not seen in their usual extra hepatic location and the common hepatic duct drains directly into the gall bladder neck with absence of the common bile duct. Further drainage of the bile away from the gallbladder and into the duodenum is provided by the cystic duct. It has also been referred to differently as the cholecystohepatic duct. Possible anomalies include congenital absence of the common bile duct, transverse lie of the gallbladder, or gallbladder interposition [1].

## Case presentation

A 46-year-old male cadaver was found to have an extra-hepatic biliary system with rare morphological anomalies. This was identified during a descriptive-prospective cross sectional study of the morphological characteristics of the extra-hepatic biliary system in humans.

The anterior abdominal wall was opened longitudinally along the midline. The abdominal wall was separated on to the right side along the central margin up to the mid axillary line. Then the abdominal wall was divided from the right side of the pubic bone up to the anterior superior iliac spine. The anterior abdominal wall flap was reflected laterally. The stomach was retracted to the left side and the second part of the duodenum, free margin of the lesser omentum, epiploic foramen and gall bladder identified. Dissection was done to demonstrate the extra-hepatic biliary system and its vascular pattern.

**Figure 1:**
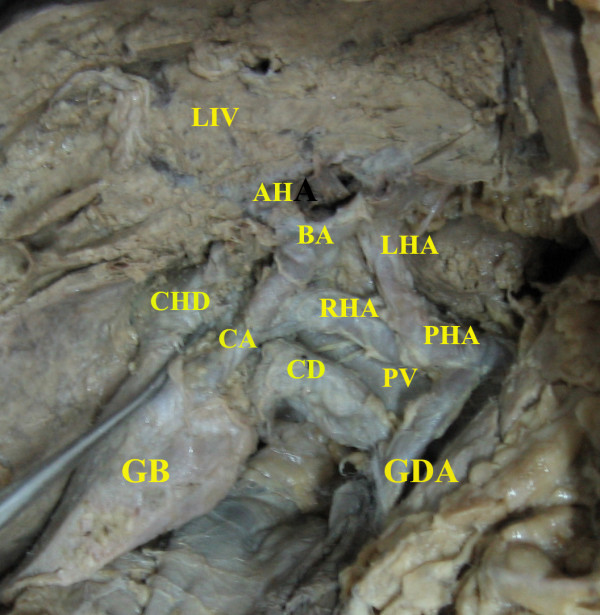
Liver (LIV) with partially dissected right lobe is seen cranially. The common hepatic duct (CHD) directly drains to the upper segment of the gall bladder (GB). From the GB the cystic duct (CD) originates and drains out as the common bile duct without joining to the CHD. The common hepatic artery is divided into the proper hepatic artery (PHA) and the gastro-duodenal artery (GDA). The portal vein (PV) is seen between the CD and the PHA. The PHA divides into left and right hepatic arteries (LHA & RHA). The cystic artery (CA) is originating from the RHA. A "bridging artery" (BA) connects the right and left hepatic arteries. An accessory hepatic artery (AH) is originating from the bridging artery, forming a "cruciate" hepatic arterial anastomosis.

The right and left hepatic ducts were not seen extra-hepatically and the common hepatic duct drained directly into the gall bladder neck, with absence of the common bile duct. Further drainage of the bile away from the gallbladder and into the duodenum was provided by the cystic duct. The gall bladder was lying in the gall bladder fossa in the right lobe of the liver in a transverse plane. The width and the length of the gall bladder were 2.5 cm and 4.5 cm respectively. The length of the common hepatic duct was 2.6 cm and the cystic duct that drained the gall bladder to the duodenum was 6.9 cm (Figure 1).

Apart from the anomalous extra-hepatic biliary morphology, a rare abnormal arterial pattern was observed. The cystic artery was anterior to the common hepatic duct and it was originating from the right hepatic artery. The division of anterior and posterior branches of the cystic artery was not noted. The right hepatic artery was found to be communicating with the left hepatic artery by a "bridging artery" after giving rise to the cystic artery. An accessory hepatic artery originated from the "bridging artery" forming a "cruciate" hepatic arterial anastomosis (Figure 1).

## Discussion

The liver, gallbladder and the extra-hepatic biliary tree arise from the hepatic diverticulum of the foregut in the beginning of the fourth week of embryological development. This diverticulum rapidly proliferates into the septum transversum and divides into two parts, the distal pars hepatica, and the proximal pars cystica [1]. At the time of appearance of the pars cystic artery, there occurs proliferation of the cells at the junction of the cystic and hepatic ducts to form the common bile duct, which is initially a cylindrical mass that undergoes vacuolation to canalize and form a single, continuous, epithelium lined lumen [1,5-7].

Failure of this normal development results in various anomalies, the rarest amongst which, is the Hepaticocystic duct. There are various patterns of Hepaticocystic ducts that have been recognized. Type I refers to the absence of the common hepatic duct where the right and left ducts drain separately into the gallbladder; Type II is when the right and left hepatic ducts unite upon entering the gallbladder; Type III refers to a common hepatic duct that enters the gallbladder, and in Type IV multiple small bile ducts connect the intrahepatic biliary system with the gallbladder. Type III is further subdivided into the common hepatic duct entering the superior wall of the gallbladder (III A), neck (III B), posterior gallbladder wall (III C), and, the fundus (III D) [8].

In our dissections the human cadaver was found to have a Type III B Hepaticocystic duct. Additionally it was found that there was no division of the common hepatic duct into right and left hepatic ducts extra-hepatically. Therefore we would like to sub-categorise our case as a variant of Type III B. There have been no previous reported cases of this nature of combined anomaly.

Although the exact etiology of this rare anomaly is unknown, it is thought to result either from failure of recanalization, with persistence of fetal communications between the gallbladder and liver [7] or from delayed division of the hepatic antrum into the cystic and hepatic diverticuli [1,2].

## Conclusion

Occurrence of a Hepaticocystic duct and an aberrant variation in the extra hepatic arterial system is extremely rare. The knowledge of such variation is important in surgical procedures related to the extra-hepatic biliary system. Comprehensive knowledge and clear visualization during surgery is mandatory for safe surgical procedures related to this important anatomical region.

## Competing interests

The author(s) declare that they have no competing interests.

## Authors' contributions

VA was responsible for the study conception and design, writing the manuscript, dissections and literature review. SS was responsible for literature review, dissection and proofreading the manuscript. KD was responsible for literature review and proofreading the manuscript. SK was responsible for literature review and dissection. All authors read and approved the final manuscript.

## Consent

Written informed consent was obtained from the patients' daughter for publication of this case report and accompanying images. A copy of the written consent is available for review by the Editor-in-Chief of this journal.
